# A Combination of Blue Light at 460 nm and H_2_O_2_ for the Safe and Effective Eradication of *Staphylococcus aureus* in an Infected Mouse Skin Abrasion Model

**DOI:** 10.3390/microorganisms11122946

**Published:** 2023-12-08

**Authors:** Vu Nguyen Ngo, Thien Nguyen Thuan Truong, Tin Trung Tran, Loan Thanh Nguyen, Ngoc Bao Mach, Van Van Vu, Thi Thu Hoai Nguyen, Thiet Minh Vu

**Affiliations:** 1 NTT Hi-Tech Institute, Nguyen Tat Thanh University, 300A Nguyen Tat Thanh, Ho Chi Minh City 70000, Vietnam; nnvu@ntt.edu.vn (V.N.N.); loannt@ntt.edu.vn (L.T.N.); mbngoc@ntt.edu.vn (N.B.M.); vanvu@ntt.edu.vn (V.V.V.); 2 School of Biotechnology, International University, Vietnam National University, Ho Chi Minh City 70000, Vietnam; tnthuanthien96@gmail.com (T.N.T.T.); ntthoai@hcmiu.edu.vn (T.T.H.N.); 3 Ho Chi Minh City University of Technology, Vietnam National University, Ho Chi Minh City 70000, Vietnam; trtrtin@hcmut.edu.vn

**Keywords:** blue light at 460 nm, hydrogen peroxide, safety, skin infection, *Staphylococcus aureus*, staphyloxanthin

## Abstract

Antibiotic-free approaches are more important than ever to address the rapidly growing problem of the antibiotic resistance crisis. The photolysis of the bacterial virulence factor staphyloxanthin using blue light at 460 nm (BL460 nm) has been found to effectively attenuate *Staphylococcus aureus* to chemical and physical agents. However, phototherapy using BL640 nm still needs to be investigated in detail for its safety in eradicating *Staphylococcus aureus* in vitro and in vivo. In this study, we employed a 460 nm continuous-wavelength LED source and a low concentration of hydrogen peroxide to treat *S. aureus* under a culturing condition and a wound abrasion mouse model. The results demonstrated the safety of the combined therapy when it did not modify the bacterial virulence factors or the susceptibility to widely used antibiotics. In addition, the results of the mouse model also showed that the combined therapy was safe to apply to mouse skin since it did not cause adverse skin irritation. More importantly, the therapy can aid in healing *S. aureus*-infected wounds with an efficacy comparable to that of the topical antibiotic Fucidin. The aforementioned findings indicate that the concurrent application of BL460 nm and hydrogen peroxide can be used safely as an alternative or adjunct to antibiotics in treating *S. aureus*-infected wounds.

## 1. Introduction

Non-antibiotic bactericidal methods, such as blue light from 405 nm to 500 nm, are of great interest due to their inherent bactericidal effect without additional external photosensitive substances [1,2,3,4]. The bactericidal mechanism of blue light is thought to be able to stimulate intrinsic photosensitizers, iron-free porphyrins, and thus generate reactive oxygen species (ROS) that are cytotoxic to bacterial cells. Recent studies show that blue light at a 460 nm wavelength (BL460 nm) can influence the cytotoxicity of *S. aureus* by disrupting the staphyloxanthin (STX) pigment structure located on the bacterial cell wall. It is postulated that STX is the virulence component that aids bacteria in their defense against oxidizing agents such as singlet oxygen (^1^O_2_), hydrogen peroxide (H_2_O_2_), peroxide anion radicals, and hydroxyl radicals (OH•) [5]. These reactive cytotoxic species can cause irreversible damage to the molecular constituents of bacterial cells. Staphyloxanthin might also have an important implication in antibiotic resistance, as the pigment involves structuring a cell wall platform for the localization and functioning of penicillin-binding protein 2a (PBP2a) encoded by the mecA gene [6]. BL460 nm is believed to be safer for human cells than blue lights at other wavelengths [7]. Therefore, the BL460 nm has a high potential for practical implementation and is the subject of ongoing research.

Previous studies have shown that using BL460 nm, whether in continuous or pulsed manners, can impart *S. aureus* to oxidizing agents to varying degrees [6,8]. Pulsed BL460 nm displays a better inhibitory effect on *S. aureus* than a continuous light source [6]. Both BL460 nm irradiance modes exhibit a strong photolysis toward STX that can be utilized to effectively eradicate the planktonic *S. aureus* and infected skin in murine models [6,9]. However, the potential limitations of pulsed light as a home device for patients stem from the increased expense and complexity associated with device assembly [10]. Numerous studies have demonstrated that using novel non-antibiotic therapies can result in unfavorable consequences that alter the biology and pathogenicity of the bacterial population being targeted [11,12,13]. Consequently, prior to implementing BL460 nm therapy in clinical settings, a comprehensive safety assessment must be undertaken.

The objective of this work is to ascertain the most effective and safe combination of BL460 nm conditions and H_2_O_2_ concentration for eliminating *S. aureus,* while ensuring that neither the bacterial pathogenicity nor the host cells are adversely affected. In this study, we also seek to establish the optimal parameter of BL460 nm for achieving efficient in vitro bacterial treatment. These factors include the manipulation of light intensities and irradiating durations at different levels. In addition, the effectiveness and safety of therapy for treating *S. aureus*-infected skin wounds are being assessed. The results of this research will help to develop a novel therapy, devoid of antibiotic usage, for treating skin infections caused by *S. aureus*.

## 2. Materials and Methods

### 2.1. Blue Light at 460 nm Source, Bacterial Strains, and Growth Condition

In a previous study, we successfully built a light-emitting diode (LED) source at a 460 nm wavelength using a COB LED LE B P2W chip (Osram Licht AG Company, Munich Germany) [14]. The LED is a chip-on-board array comprising 216 LED chips per square centimeter. To generate an appropriate illumination field, we used a converging lens made of glass with a light transmission at 460 nm > 90% and a focal length of 50 mm, positioned approximately 50 mm away from the LED array. The LED was calibrated to emit 460 nm wavelengths with a narrow spectral peak and minimal heat emission. The BL460 nm emitted via the LED system was adjusted to the desired light intensity at specific cross-section areas using a built-in pulse-width modulation (PWM) compartment, calibrated using a spectroradiometer (Apogee Instruments, Logan, UT, USA). The PWM controls the average power delivered by the electrical signal of the P2W chip, allowing the light intensity to be increased and decreased via an adjustable dimmer switch.

The electrical power of the LED is 60 W (adjustable via a current source), but its luminous efficacy is approximately 50%, meaning about 30 W of light energy. The illuminated field used in the study has a circular shape with a diameter of 8 cm (equivalent to an area of approximately 50 cm^2^), resulting in a maximum light energy density of 600 mW/cm^2^. The exact light intensities (50, 100, 200, and 400 mW/cm^2^) at specific cross-section areas were adjusted and calibrated with the help of a spectroradiometer (Apogee Instruments, USA). All irradiation treatments were performed at a room temperature of 25 °C. 

The strains of *S. aureus* used in the study were the methicillin-resistant *Staphylococcus aureus* (MRSA) strain ATCC^®^ 33592^TM^ and methicillin-susceptible *Staphylococcus aureus* (MSSA) strain ATCC^®^ 29213™ (American Type Culture Collection, Manass, VA, USA). The bacterial cells were grown and stocked in a Trypticase soy broth (TSB) medium (Oxoid, Thermo-Fisher Scientific, Altrincham, UK).

### 2.2. The Growth of the S. aureus under the BL460 nm Irradiance

**Figure 1:**
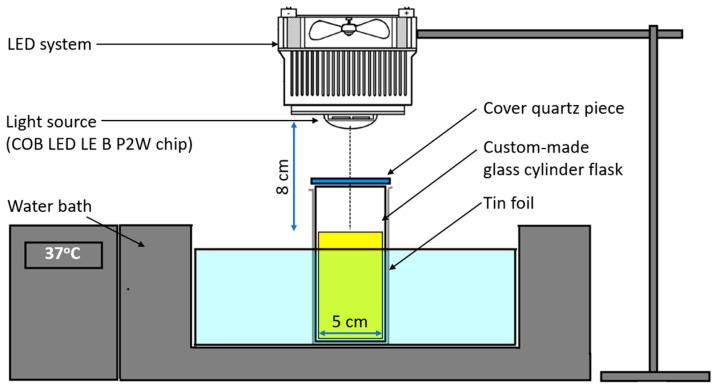
The experimental setup of sample irradiation process.

The MSSA and MRSA cells were grown in a tryptic soy broth (TSB) medium at 37 °C to a suspension of approximately 10^8^ CFU/mL. The suspension was then inoculated into 150 mL of the fresh TSB medium and cultured to a 10^5^–10^6^ CFU/mL density. The entire volume of the culture was subsequently transferred to a cylindrical glass flask with a bottom diameter of 5 cm; the mouth of the flask was covered with a round piece of quartz to prevent contamination during the light treatment process. The flask was incubated at 37 °C under the following BL460 irradiating conditions (Figure 1).

To ensure uniform illumination over the sample area, we avoided a direct focus on the lens focal point, which would concentrate light at the LED points. Instead, the sample was positioned about 8 to 10 cm away from the lens. This arrangement allowed light from the LEDs in the chip-on-board LED array to interfere with each other, promoting a more even and broader illumination field suitable for the experiment. The cross-section area produced by the LED system was a circular shape, 3 cm in diameter when measured directly after the source. The cross-section area was almost unchanged (approximately 3 cm) at 8 cm above the bacterial suspension (Figure 1 and Supplemental Figure S1). All irradiated samples were positioned at this constant distance from the LED light to minimize the effects of focal length variation on light intensity. The LED light positioned 8 cm above the flask was operated to emit the BL460 nm directly into the bacterial suspension.

### 2.3. Virulence and Antibiotic Susceptibility Testing

*S. aureus* was exposed to BL460 nm irradiance to test the production of caseinase for casein hydrolysis virulence [15], gelatinase for hydrolysis of the host extracellular matrix [16], lipase for lipid hydrolysis [17], lecithinase for cytotoxicity to animal tissue [18], and hemolysin [19]. A bacterial inoculum was prepared by resuspending overnight-grown *S. aureus* cells in PBS to 10^6^ CFU/mL. A 5 mL cell suspension volume was transferred to cylindrical glass flasks and treated with different intensities of BL460 nm (50 mW/cm^2^, 100 mW/cm^2^, 200 mW/cm^2^, and 400 mW/cm^2^). The bacteria were collected 5 min, 10 min, 20 min, and 40 min after irradiance. The bacteria suspension (10 µL) was added to skim milk, gelatin, tributyrin, tryptic soy, and sheep blood agars for caseinase, gelatinase, lipase, lecithinase, and hemolysin tests, respectively. The material and substrate medium were obtained from Oxoid (UK). The agar plates were then incubated at 37 °C for 24–48 h. After incubation, the enzymatic hydrolysis zones surrounding the bacterial colony spot were measured in millimeters. The activity of each virulence factor was evaluated by calculating the Enzyme activity Index (EAI) = (Diameter of hydrolysis zone)/(Diameter of bacterial colony spot). An EAI = 1 was considered as no change in that tested virulence factor [20].

The antibiotic susceptibility of the MRSA strain was evaluated by using the antibiotic paper disc diffusion method following the European Committee on Antimicrobial Susceptibility Testing—EUCAST [21]. The MRSA colonies were resuspended in a Mueller–Hinton Broth (MHB) medium to prepare a bacterial suspension of approximately 10^6^ CFU/mL. In cylindrical glass flasks, a 5 mL bacterial suspension was illuminated with BL460 nm for 10 min, 20 min, and 40 min. After light treatment, the cell suspension was spread on a 4 mm thick Mueller–Hinton Agar (MHA) plate. Subsequently, Antimicrobial Susceptibility Discs (Oxoid, Altrincham, UK) were placed on the surface of the agar. After incubating the MHA plates at 37 °C for 18–24 h, the inhibitory zone diameter was determined in millimeters. The changes in antibiotic susceptibility were calculated using the zone of inhibition change index = (zone of inhibition of light-treated MRSA)/(zone of inhibition of negative control MRSA). The zone of inhibition change indexes of all 21 antibiotics were illustrated in a heatmap using GraphPad PRISM 9.5.1 (San Diego, CA, USA, www.graphpad.com, accessed on 8 February 2023).

### 2.4. Anti-MRSA Assay with BL460 nm in Combination with H_2_O_2_

Similar to the previous tests, cylindrical glass flasks containing 10^7^ CFU/mL of the MRSA suspension were illuminated with BL460 nm at 50 mW/cm^2^, 100 mW/cm^2^, 200 mW/cm^2^, and 400 mW/cm^2^ for 5 min, 10 min, 20 min, and 40 min. A volume of 100 µL of each light-treated cell suspension was added to the wells of a 96-well plate which were pre-filled with 100 µL of a two-fold serial dilution of H_2_O_2_, ranging from 3% to 0.08%. After 5 min of exposure to H_2_O_2_, each bacterial mixture was removed and spread on the TSA plate so that the surviving bacterial cells could grow and form colonies. The minimum inhibitory concentration of H_2_O_2_ (MIC_H2O2_) was determined to be the concentration of H_2_O_2_ at which all MRSA cells were killed. The number of colonies on the plate after 24 h of incubation was also used to calculate the bacterial density (CFU/mL).

### 2.5. Safety of 460 nm Light and H_2_O_2_ on Bare Skin

A mouse skin model was tested for the safety of using the 460 nm and H_2_O_2_ treatment. Ten mice (*Mus musculus* Swiss Albino) had their back hair shaved to expose a 2 × 2 cm skin area. The light beam of BL460 nm was adjusted to cover the skin at the 100 mW/cm^2^ intensity for 5 min, followed by the application of a 0.75% H_2_O_2_ solution once daily for 10 days. Daily observations and photographs were taken of the treated skin in order to detect signs of skin irritation, such as discoloration, dermatitis, or redness.

### 2.6. MRSA-Infected Wound Model

Seven–eight-week-old mice were housed for one week prior to use in the experiment. Each individual was housed in a separate cage and fed rice bran pellets. The MRSA infection mouse model was induced by injecting cyclophosphamide, as described in previous studies [22], and creating an excisional infection wound following procedures described previously elsewhere [13,23]. Before the surgery to create an infected wound, each of the ten mice (five males and five females) were injected with cyclophosphamide (Sigma-Aldrich—C0768) at doses of 150 mg/kg, 250 mg/kg, and 350 mg/kg. Each dose of cyclophosphamide was divided into two smaller doses and administered intravenously four and one day before the surgery.

### 2.7. A Mouse Skin Abrasion Model Infected with S. aureus

The cyclophosphamide-treated mice were anesthetized via intraperitoneal injection with a mixed dose of 5 mg/kg of Zoletil and 4 mg/kg of Xylaxin (Virbac, Paris, France). Subsequently, the back of each mouse was razor-shaved to expose a 3 × 3 cm skin region. Then, a 1 cm diameter circular portion of the dorsal skin was removed using sterile surgical scissors and forceps. The cutting had to remove the epidermis, dermis, and subcutaneous layers to expose the underlying muscle [24]. The open muscle was then lightly abraded with a scalpel. Immediately after the abrasion, one drop of the 10 μL (10^8^ CFU/mL) MRSA bacterial suspension was applied to the wound. The wound was immediately covered with 2 × 2 cm of Tegaderm™ tape (3M, Hochiminh city, Vietnam), and a sterile medical bandage was wrapped around the mouse’s waist. The mice recovered in their cage for one day before the bandages were removed. The mouse mortality in each cyclophosphamide-treated group was monitored for ten days. Based on the survival rate and infection efficiency, the optimal cyclophosphamide dose for the mouse infection model was determined.

### 2.8. Treatment of MRSA Infection Wound Model Using 460 nm Light and H_2_O_2_ Combination

Mice with successfully infected wounds were divided into five groups of 20 individuals each. Each group received a unique treatment for the injured back skin. The group 1 mice were untreated, serving as a negative control. The group 2 mice were administered 0.75% H_2_O_2_. The group 3 mice were exposed to 460 nm light for 5 min at 100 mW/cm^2^. As a positive control, group 4 mice were topically treated with Fucidin H^®^ cream, which contains the antibiotic fusidic acid and Hydrocortisone specifically for skin infections caused by *Staphylococcus* bacteria. The group 5 mice received BL460 nm and H_2_O_2_. Individual mice in groups 3 and 5 were placed in transparent polyethylene (PE) plastic boxes to restrict the animals’ mobility during treatment. The mouse wound was illuminated vertically with the BL460 nm LED source at a 10 cm distance. At this distance, the area of the lesion skin lies within the light’s cross-section area. The group 5 mice received 0.75% H_2_O_2_ on their lesions after light treatment. Every mouse in each group received treatments daily for fifteen consecutive days.

The infected wound was photographed daily throughout the course of the treatment. The ImageJ 1.53k software (NIH, Bethesda, MD, USA) was used to measure and quantify the size of the wounds’ surface areas. To assess the wound-healing rate across treatment groups, the wound area was converted into the percentage of wound healing (%WHL) value, which equals (1-W1/W0) × 100 [13]. W1 is the wound area of the individual mouse on the date of recording, and W0 is the initial wound area on the first day after bandage removal.

### 2.9. Statistics

A statistical analysis was performed using GraphPad PRISM 9.5.2, in which the normality of the data was analyzed using an integrated Shapiro–Wilk test. All replicated data sets are presented as the mean ± SEM or SD, with differences between means compared for significance using either Student’s *t*-test or a one-way ANOVA, following appropriate post hoc testing for pairwise comparisons. *p* values of less than 0.05 were considered significant.

## 3. Results and Discussion

### 3.1. Blue Light at 460 nm Delayed the Lag Phase during the Growth of the Bacteria

We designed and assembled a 460 nm wavelength-emitting LED system as previously described [14]. Using this LED setup, we demonstrated that the BL460 nm effectively photolyzed the carotenoid pigment STX isolated from *S. aureus*. The BL460 additionally showed the capacity to directly photolyze the color of the STX pigment on the MSSA and MRSA cells but did not elicit antibacterial effects [14]. In this study, we evaluated the effect of BL460 nm on the biological and physiological properties of *S. aureus*. 

**Figure 2:**
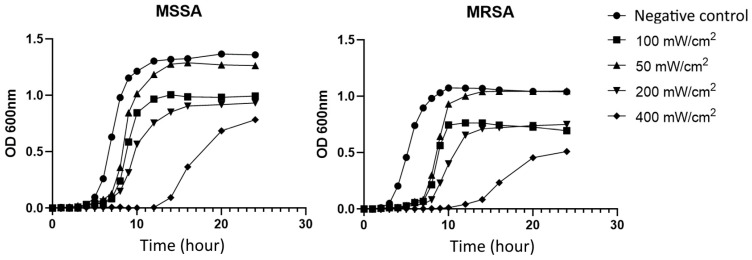
BL460 nm delays the growth of *S. aureus* in the lag phase. Both the MSSA and MRSA were cultured under continuous BL460 nm at different light intensities. Each data point expresses the mean of three technical measurements of OD600.

The MSSA and MRSA cells were grown under BL460 nm at four light intensities (50, 100, 200, and 400 mW/cm^2^) measured at the cross-section areas. The growth curves clearly showed that the BL460 nm light significantly hindered the growth of the bacteria. Specifically, *S. aureus* cells considerably prolonged the lag phase (Figure 2). Under 400 mW/cm^2^, the lag phase of the studied MSSA and MRSA strains lasted up to ten hours. In contrast, the untreated cells completed the lag phase in four hours, and the cells entered exponential growth. Interestingly, the delay in the lag phase for the MRSA was more profound than for the MSSA. Compared to the untreated cells and the 50 mW/cm^2^ light-treated cells, cells growing at 100, 200, and 400 mW/cm^2^ achieved a plateau at lower OD600 values. These results indicate that BL460 nm at a higher intensity strongly affected the biology of *S. aureus* during the lag phase, which may lead to intrinsic changes in the biological properties of the bacterial cells. The fact that the light-treated cells had lower OD600 values for plateau implies that the light might also have a deleterious impact on the growth of *S. aureus* during the lag and(/or) exponential phases.

### 3.2. BL460 nm Does Not Modulate the Pathophysiological Adverse Properties of the Tested S. aureus

Next, we seek to know to what extent BL460 nm influences the pathophysiological characteristics of *S. aureus*, including its virulence and sensitivity to antibiotics. The changes in virulence factors were evaluated in the bacterial cells after being treated with various BL460 nm doses, which can be tracked based on the duration and intensity of irradiance. The cells were collected at four different irradiating durations (5, 10, 20, and 40 min) for each of the four light intensities used (50, 100, 200, and 400 mW/cm^2^).

**Figure 3:**
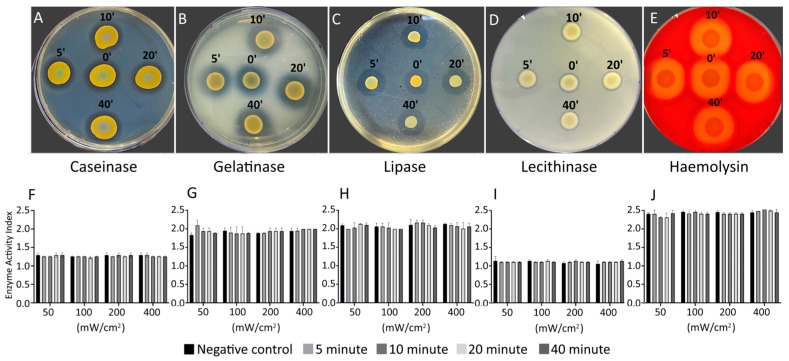
Evaluation of virulence factors of MRSA under BL460 nm irradiance. The changes in virulent factors of the bacterial cells were examined under different light intensities (50, 100, 200, and 400 mW/cm^2^) and different light treatment durations (5, 10, 20, and 40 min). (**A**,**F**): Caseinase. (**B**,**G**): Gelatinase. (**C**,**H**): Lipase. (**D**,**I**): Lecithinase D. (**E**,**J**): Hemolysin. Graphs in (**F**,**J**) were plotted from data of four replicates. Data are expressed as mean and standard deviation (SD). No statistical significance was found from the results of ANOVA and post hoc Tukey analysis for all the data.

The data demonstrated that the MRSA strain exposed to BL460 nm did not differ from the untreated cells in any of the virulence factors evaluated, including casein hydrolysis virulence, gelatinase, lipid hydrolysis, lecithinase, and hemolysis (Figure 3). This finding suggests that the BL460 nm did not alter the MRSA’s capacity to manufacture any of the assessed virulence factors. Therefore, we conclude that BL460 nm may be employed without endangering *S. aureus* or resulting in a predisposed increase in bacterial toxicity. We obtained similar results with the MSSA strain (Supplemental Figure S2).

### 3.3. Changes in the Antibiotic Susceptibility of S. aureus under BL460 nm Irradiance

**Figure 4:**
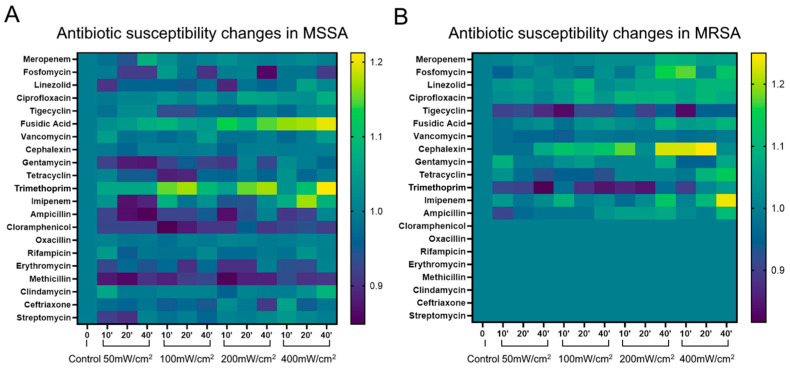
Evaluation of antibiotic susceptibility of MSSA and MRSA strains after BL460 nm exposure. Heat maps illustrate the ratio values of the zone of inhibition change index. Ratio values > 1 (green to yellow) indicate increased antibiotic resistance. A ratio value < 1 (dark blue to purple–black) indicates a decrease in antibiotic resistance. A ratio value of approximately = 1 (blue) indicates no change in antibiotic resistance. (**A**) Changes in antibiotic susceptibility in MSSA and (**B**) in MRSA. The ratio calculation was presented in the method section.

When bacteria are exposed to non-antibiotic antimicrobial agents, new antibiotic resistance can evolve or become more pronounced in some strains of bacteria. These unanticipated adjustments may restrict the use of novel antimicrobial therapies. Therefore, in this study, we examined how the BL460 nm irradiance affects the drug susceptibility of *S. aureus*. We found that, among the 21 antibiotics used, the light-treated MSSA strain tended to be more sensitive to the antibiotics fusidic acid and Trimethoprim. This increased sensitivity was clearly shown when the cells were exposed to 400 mW/cm^2^ of BL460 nm for 40 min (Figure 4A).

BL460 nm exposure also showed the enhanced resistance of the MSSA strain to Chloramphenicol, Gentamycin, and Methicillin. Nevertheless, the observed changes were statistically insignificant, as the diameter of the zone of inhibition exhibited only marginal fluctuations ranging from 1 to 2 mm. The resistance to the remaining antibiotics varies unevenly (Figure 4A).

The light-treated MRSA strain showed increased susceptibility to the antibiotics Fosfomycin, Cefalexin, and Imipenem, as evidenced by an increase in the antibacterial zone diameter from 3 to 5 mm. This increase in antibiotic sensitivity was clearly shown in the cells treated with 400 mW/cm^2^ of light intensity for 40 min (Figure 4B). Several other antibiotics, including Meropenem, Linezolid, Ciprofloxacin, fusidic acid, Tetracycline, and Ampicillin, exhibited a slight increase in antimicrobial efficacy. Similar to the MSSA strain, the MRSA strain treated with BL460 nm did not show a noticeable enhancement in its antibiotic resistance. Overall, the aforementioned data indicate that the utilization of BL460 nm did not result in a significant elevation in resistance against the majority of antibiotics. Hence, the light can be used safely with most antibiotics without causing adverse consequences.

### 3.4. BL460 nm Decreases Minimum Inhibitor Concentration Value of H_2_O_2_

Our work and other studies show that *S. aureus* cells could not be killed with BL460 nm via the photolyzing of the STX pigment. However, the light attenuated the *S. aureus* to a low concentration of H_2_O_2_ in culturing conditions and an infected skin lesion in a murine model. In this study, we employed a similar approach to determine the minimum concentration of H_2_O_2_ that can be paired with the LED 460 nm system to eradicate *S. aureus* cells. To identify the ideal lighting setup suitable for practical and clinical settings, we screened for various BL460 nm doses, considering both the light intensity and the duration of irradiation.

**Figure 5:**
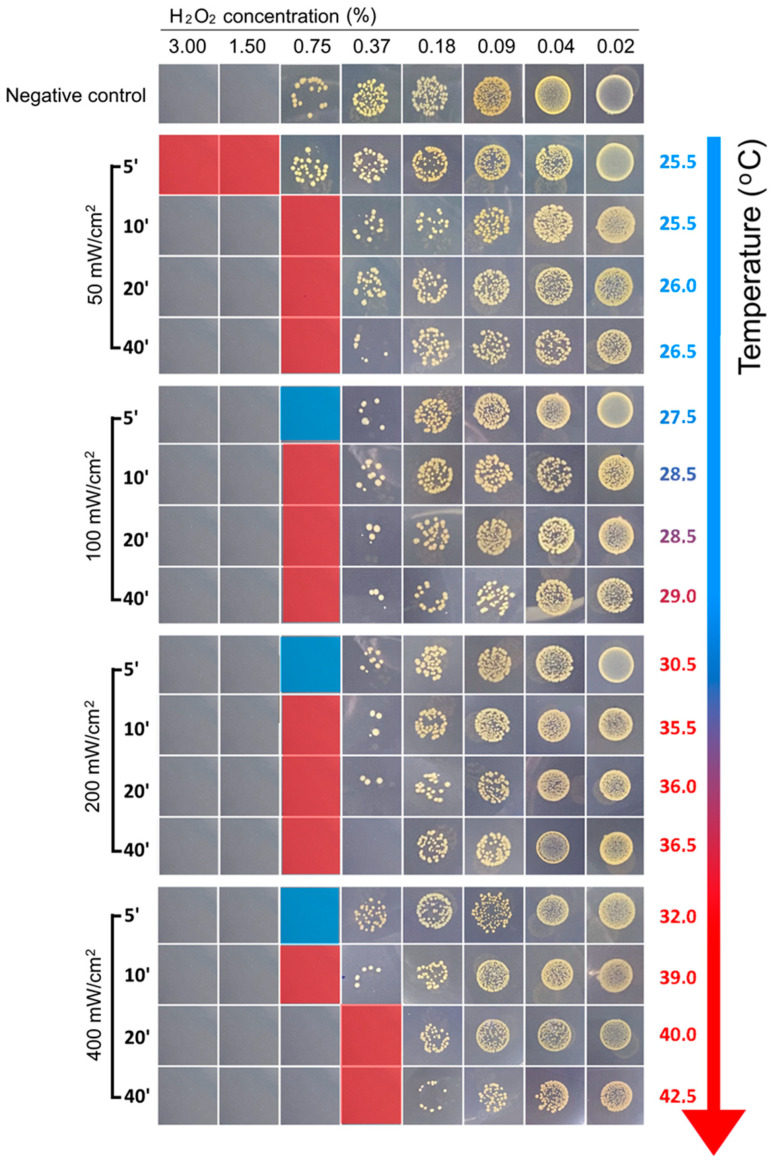
The changes in hydrogen peroxide sensitivity of the MRSA after BL460 nm irradiance. The bacterial cells were tested for the MIC_H2O2_ value under different BL460 nm intensities and treatment durations. The temperature of the irradiated samples was monitored during the treatment. Squares with red labeling indicate the values of MIC_H2O2_ for each experimental condition.

We first determined the MIC value of H_2_O_2_ (MIC_H2O2_) for the MRSA 33592TM strain when subjected to BL460 nm treatment. The results showed that, with an intensity of 50 mW/cm^2^, it took 10 min of light treatment to obtain an MIC_H2O2_ of 0.75% compared to the MIC_H2O2_ of 1.5% in the untreated bacterial cells (Figure 5). Meanwhile, it only required 5 min to elicit the same impact of lowering the MIC_H2O2_ when 100 mW/cm^2^ and higher light intensities were employed to treat the bacterial cells (Figure 5).

We noticed that the MIC_H2O2_ for the MRSA did not alter significantly at the three light intensities of 100, 200, and 400 mW/cm^2^. When the bacteria were illuminated for 20 min at an intensity of 400 mW/cm^2^, the MIC_H2O2_ could decrease to 0.375%. Nevertheless, when exposed to the light intensities of 200 and 400 mW/cm^2^, the temperature of the samples illuminated for 20 min increased by more than 30 °C and 40 °C, respectively. Therefore, it is impracticable to use intensities of 200 and 400 mW/cm^2^ in animal models and clinical contexts due to the elevated temperature of the illuminated sample. Given the aforementioned heating concern, we deduced that the optimal conditions for the forthcoming in vivo experiment are the combination of a 100 mW/cm^2^ light intensity for 5 min and a H_2_O_2_ concentration of 0.75%.

### 3.5. Combination of BL460 nm and Hydrogen Peroxide Is Safe for the Skin

**Figure 6:**
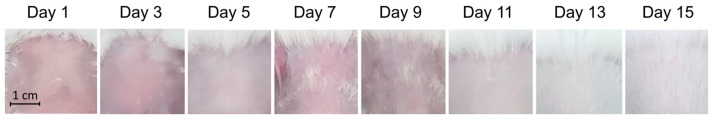
The test of skin sensitivity treated with the combined therapy during 15-day treatment. A skin area of 2 × 2 cm was exposed to BL460 nm at 100 mW/cm^2^ in 5 min, and was then swiped with 0.75% H_2_O_2_. The whole procedure was repeated daily for 15 days. The treated skin area was pictured and recorded for rashes, changes in color, and skin irritation caused by the combined therapy.

Next, we investigated the antibacterial effects of combined therapy (BL460 nm at 100 mW/cm^2^ for 5 min and 0.75% H_2_O_2_) on a mouse wound infected with MRSA. We first assessed the safety of the combined therapy on mouse skin. A 2 × 2 cm area of open dorsal skin was exposed to 100 mW/cm^2^ of BL460 nm for 5 min, after which the skin was wiped with the 0.75% H_2_O_2_ solution. The procedure was carried out on the targeted skin area every day for 15 days. There were no visible rashes or other adverse effects on the epidermis, as shown in Figure 6. Therefore, we conclude that it is safe to apply the combined therapy to mouse skin.

### 3.6. Combined Therapy Showed Skin Abrasion Healing Comparable to Fucidin

**Figure 7:**
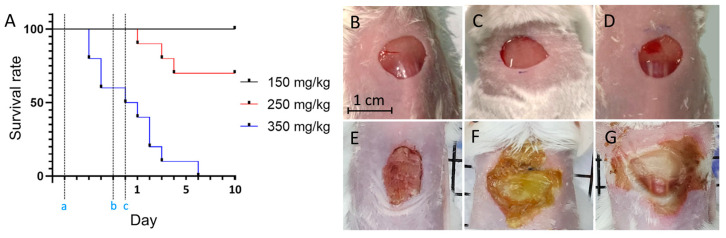
Survival analysis of mice who underwent different doses of cyclophosphamide injection. (**A**) The symbol “a” indicates the day of the first treatment, “b” indicates the second cyclophosphamide treatment, and “c” indicates the day of *S. aureus* being infected on the skin. (**B**–**D**) The open skin sites after the second dose of cyclophosphamide injection and the initial application of MRSA. (**E**,**F**) The injured skin area after five days of cyclophosphamide treatment and MRSA infection; (**B**,**E**): 150 mg/kg cyclophosphamide, (**C**,**F**): 250 mg/kg cyclophosphamide, (**D**,**G**): 350 mg/kg cyclophosphamide.

We then examined the therapeutic efficacy of BL460 nm and H_2_O_2_ on a mouse skin abrasion model infected with *S. aureus*. The mice were first subjected to immuno-suppression via an IP injection of cyclophosphamide to ameliorate the infection. The appropriate amount of the drug was determined by testing with three concentrations of cyclophosphamide (50, 100, and 250 mg/kg of body weight). The 50 mg/kg dosage of cyclophosphamide was insufficient to induce the infection caused by MRSA, as indicated by the self-healing of the infected skin (Figure 7B,E).

The mice injected with 250 mg/kg of cyclophosphamide had a 70% survival rate on day four and lasted until the end of the 10-day trial (Figure 7A). The wounds in this group showed signs of spreading compared to the original, accompanied by a yellow pus appearance and swollen wound edges on the first day, demonstrating that the infectious process of the MRSA bacteria in the mice was successful (Figure 7C,F). The mice that survived to the 10th day showed recovery, and the wound began to close. The mice injected with 350 mg/kg of cyclophosphamide had the lowest survival rate, with 40% of the mice dying three days after the first injection (200 mg/kg) and only 50% of the mice surviving after the second injection (Figure 7A, blue line). The infected wounds of the animals in this group that survived until day 5 showed severe infection. The infection began to spread beneath the skin and ran along the trunk and body (Figure 7D,E). We therefore conclude that the dose of 250 mg/kg is most suitable to induce skin abrasion with MRSA infection in Swiss albino mice.

### 3.7. Low Concentration of H_2_O_2_ and BL460 nm Combination Effectively Heal the Skin

Next, we sought to evaluate the efficacy of the combined therapy of BL460 nm and H_2_O_2_ in promoting healing in the skin-infected mouse model. The mice with skin abrasion infected with MRSA were subjected to five different regimens, designated as group 1 to group 5, as described in the Materials and Methods Section.

**Figure 8:**
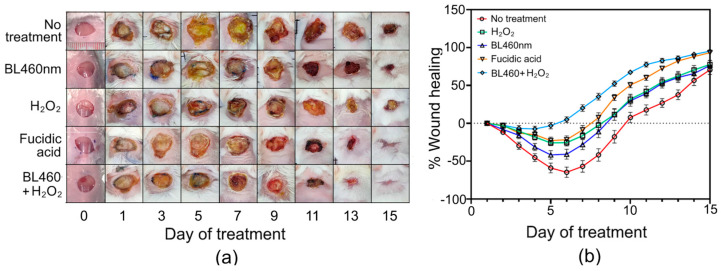
Wound-healing recovery of an abrasion skin infection model. (**a**) The skin-healing model in mice was treated with only BL460 nm, H_2_O_2_, fusidic acid and the combination of BL460nm and H_2_O_2_. Day 1 indicates the initiation of treatment. (**b**) Wound healing after 15 days of different treatments was recorded on a daily basis. Data are expressed as mean and standard error of the mean (SEM) of at least 10 animals per group.

Group 1 (no treatment) displayed the worst wound-healing progression among the five groups. In the first three days, the wound area of the mice in group 1 grew by up to 30% (±4.72%) of the original wound size, and the infection peaked on day six, when the wound area grew by over 64.5% (±6.9%). On day seven, the healing process of group 1 was observed when the wound started to desiccate and constrict (Figure 8a,b). On the final day of the experiment (day 15), only three out of the initial group of 20 untreated mice had fully healed wounds. The remaining 17 mice still had wounds that had not yet recovered. The average wound-healing length (WHL) value for these mice was 70.47% (±6.04%).

**Figure 9:**
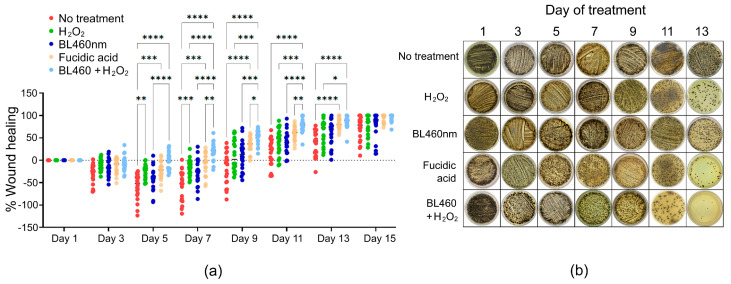
Statistical analysis of the healing efficacy of different therapies in the skin model and its correlation with the number of MRSA colonies on the skin lesion. (**a**) Statistically significant differences among the treated groups of animals. Each circular point represents data from one animal. (**b**) Image of MRSA colonies collected from the wounds when smeared and cultured on Baird-Parker agar. The one-way ANOVA and Tukey post hoc test were used to determine the statistically significant comparison between the two groups. Those pairs that show statistical significance are highlighted. *: *p *< 0.0332; **: *p* < 0.0021; ***: *p* < 0.0002; ****: *p *< 0.0001. No asterisk between groups means *p* > 0.05. Each point in the data represents an individual animal. Normality of data was checked via Shapiro–Wilk test in GraphPad Prism (Supplemental Table S1 and Supplemental Figure S3).

The group 2 mice that were illuminated with BL460 nm for 5 min per day had comparable wound healing to the group 1 mice for the first three days (*p* > 0.05) (Figure 9a). On the fifth day, a notable disparity in the wound-healing process was observed between group 2 and group 1. Specifically, the wound area of the group 2 mice only expanded by 41.76% (±5.68%) compared to 58.94% (±6.54%) in group 1. In addition, the wounds of the group 2 animals also began to show signs of healing. The statistical analysis showed that there was a significant difference in the %WHL of the mice between groups 1 and 2 on day 5 (*p* < 0.0021) and day 7 (*p* < 0.0002) (Figure 9a). Notably, by the ninth day of the treatments, the rate of wound healing in group 1 was lower than in group 2. On this day, the average percent WHL value of group 1 on this day was −17% (±8.32%), compared to 11% (±7.25%) in group 2. The statistical analysis, however, revealed no significant difference between these two groups (Figure 9a).

In groups 3 and 4, the mice were treated with H_2_O_2_ and Fucidin, respectively. In comparison to group 1 and 2, the wounds treated with the two bactericidal agents had a striking effect on the first day. Groups 3 and 4 healed at comparable rates for the first six days; however, beginning on day 7, the Fucidin-treated group experienced a more rapidly reduced wound size than the H_2_O_2_-treated group. On the final day of treatment (15 days), seven of the 20 mice in the H_2_O_2_-treated group were completely cured. In the Fucidin-treated group, 11 of the 20 mice were completely healed, with three individuals recovering entirely by day 13 (Figure 8). The statistical analysis, however, displayed no significant difference between groups 3 and 4 during the 15-day experiment (*p* > 0.05) (Figure 9a).

Group 5, which utilized BL460 nm and H_2_O_2_ to treat the wounds, demonstrated outstanding outcomes compared to groups 1, 2, 3, and 4 (*p* < 0.0001), particularly within the first week of treatment (Figure 9a). The mice in group 5 had the quickest wound-healing rates. Even though the infected wound continued to expand during the initial five days of treatment, group 5 exhibited the lowest mean percent WHL value at −7.47% (±3.57%), indicating that the wound of this group grew by no more than 7.47% of its initial wounded size (Figure 7B and Figure 8a). These findings suggest that the combination of BL460 nm and H_2_O_2_ potentially inhibited the wounds’ expansion, thus accelerating the healing process. In addition, the lesions in group 5 were dry and rarely showed yellow pus discharge. In particular, the wound surface developed dry scabs quickly, on average, within 6 to 7 days of treatment (Figure 8a). Thus, combining BL460 nm with an intensity of 100 mW/cm^2^ for 5 min with a 0.75% H_2_O_2_ solution was as effective as using Fucidin in treating MRSA-infected wounds.

### 3.8. Combined Therapy Might Eradicate S. aureus More Effectively Than Fucidin

To track the number of bacteria during the course of treatments among the five groups, smears of the skin lesion area were collected and cultured on Baird-Paker agar. The formation of colonies was observed and recorded. The data indicated that the lowest number of colonies were found in group 5, indicating that the combined method was more successful than fusidic acid at eliminating the bacteria from the skin lesion (Figure 9b and Figure S4).

## 4. Discussion

In this study, we illustrated that using BL460 nm with a low concentration of H_2_O_2_ (0.75%) in eradicating *S. aureus* from a skin abrasion in mice was safe and effective. We demonstrated the safety of BL460 in the context of virulence toxicity and antibiotic resistance. Upon BL460 nm treatment, neither the MRSA nor the MSSA in this study were affected in terms of virulence production. The BL460 nm had a negligible influence on the development of antibiotic resistance in the MRSA and MSSA strains studied. In addition, a 10-day evaluation of the combined therapy on the skin of the mice revealed no adverse effects, indicating that BL460 nm and H_2_O_2_ are safe for the skin. We also demonstrated the therapy’s efficacy in treating *S. aureus*-infected skin wounds in mice. The effect of the combined method on wound healing was comparable to that of the antibiotic Fucidin. Intriguingly, the combined therapy reduced the number of *S. aureus* cells in the wound more effectively than Fucidin.

Using lights at various wavelengths is of great interest for developing non-antibiotic bactericidal therapies [4,25,26]. BL460 nm was selected due to its advantages in host cell safety compared to other wavelengths. Multiple studies have confirmed that BL460 nm is insufficient for killing bacteria. However, BL460 can cause detrimental changes in bacteria, rendering them susceptible to destruction by adjuvant agents (such as magnetic induction, microwaves, and silver nanoparticles) [27,28]. Recent studies showed that BL460 nm effectively photolyzes the STX pigment on the *S. aureus* cell wall. This pigment functions as a virulence factor, assisting bacterial cells in resisting oxidative stresses generated by host cells [5,29]. STX is also a crucial component of the microdomains that aid in the precise localization of cell wall proteins, including those that bind penicillin [6]. Therefore, the application of BL460 nm treatment elevates the vulnerability of bacterial cells to various conventional antibiotics [6].

Our findings align with the study by Dong et al. (2021), in which they used pulse light at BL460 nm and a lower H_2_O_2_ concentration of 0.35% to kill *S. aureus* [6]. They also showed that the pulse laser’s 460 nm irradiance effectively damages the membrane microdomain structure, which STX stabilizes. The disorganization of the microdomain was also shown to disrupt the function of penicillin-binding protein 2, rendering the bacterial cells more vulnerable to penicillin and other beta-lactam-based antibiotics. Our study used continuous BL460 mm irradiance, which is more common and less expensive to treat skin infected with *S. aureus* when the light is combined with a small amount of H_2_O_2_. While the pulsed light demonstrated better efficacy than continuous light in eradicating several bacterial pathogens, the complexity of pulsed light generation may limit its potential clinical application [25,26,27,28,30]. Therefore, future research should concentrate on developing a cost-effective and straightforward home device that utilizes continuous illumination.

Furthermore, we investigated the light dose in detail for the intensity and irradiation duration required to treat *S. aureus* effectively. This information is beneficial for those who want to use the same concept to design their own method of utilizing BL460 nm to eradicate *S. aureus*. For therapeutic effectiveness, we recommend that 100 mW/cm^2^ irradiance for 5 min should be used. The 5 min irradiation is also practical for patients. The length of therapy is crucial for clinical applications because it is not empirical if the irradiating period is too long, which can cause several problems (e.g., overheating).

Nonetheless, these results should be interpreted with caution, and several limitations should be considered. The concern of resistance development in the bacteria to cope with the deleterious action of BL460 nm has been tested previously. In one study, after light treatment, the bacterial cells were sub-cultured for ten generations and then tested for drug resistance [6]. The findings indicated that BL460 nm did not induce sustainable resistance to all tested antibiotics. The explanation that was proposed was because the light has non-specific and broad impacts on multiple targets, such STX and other cellular targets, that can impair the survival function of *S. aureus*.

Our study exclusively examined the effects of a single light exposure on the virulence and antibiotic resistance of the bacteria. It is therefore difficult to say if phototherapy is safe when used more frequently. Due to the limited sample size of two *S. aureus* ATCC strains, it is difficult to generalize these findings to various strains. Extensive research on other strains of *S. aureus* will be needed to show how well the combination therapy works, which will help to build strong evidence that the treatment could be used clinically to treat skin lesions infected with *S. aureus*. Despite this, a multitude of studies have provided the evidence that blue light is safe for both bacterial and host cells [1,13,25,31,32].

In the antibiotic susceptibility testing, we observed that the MSSA became more susceptible to Fucidin. The increased sensitivity of bacteria to fusidic acid under blue light treatment was also confirmed in another study [33]. Topical Fucidin is still prevalent in treating skin lesions infected with *S. aureus*. Therefore, the combined therapy should be investigated for use with fusidic acid to enhance the efficacy of the drug and potentially diminish the MIC of fusidic acid, thereby mitigating the occurrence of fusidic acid resistance. The 460 nm light does not make *S. aureus* bacteria more dangerous; on the contrary, it also weakens this bacterium to certain antibiotics, especially making MRSA bacteria more sensitive to Imipenem and Ampicillin, the two antibiotics that are less effective against MRSA. The improvement of the bactericidal efficacy of antibiotics via BL460 nm should also be interesting to explore in the future.

In order to advance the application of BL460 nm and H_2_O_2_ to human subjects, it is imperative that the combined approach be evaluated on a larger sample size, encompassing a wider range of infected lesions (e.g., postoperative, diabetes mellitus). To ensure that the combined therapy has a wider antibacterial spectrum of action, it is necessary to examine additional *S. aureus* strains with distinct antibiotic resistance spectra. The combined method is also needed to test for the Panton–Valentine leucocidin-producing MRSA positive strain (PVL+ strain), as it is highly associated with skin and soft tissue infection diseases [34,35]. It would be beneficial to apply the light and H_2_O_2_ methods to treat the PVL+ strain, which is highly prevalent in a community-acquired manner [36,37]. In summary, further research is required to establish the effectiveness of BL460-based therapy for application in humans.

## 5. Conclusions

*S. aureus* is a significant human pathogen that causes infections in clinical settings and communities. MRSA variants in particular play a key role in infection. However, existing antibiotic development pipelines have been falling behind the rate at which new resistant bacterial strains emerge [38,39]. The 460 nm light combined with H_2_O_2_ presented in this study will provide a potential non-antibiotic therapy which is efficient and safe for killing *S. aureus* in both in vitro and in vivo models.

## Data Availability

The data supporting the reported results can be provided upon a written request to the corresponding author.

## Supplementary Materials

The following supporting information can be downloaded at https://www.mdpi.com/article/10.3390/microorganisms11122946/s1, Figure S1: The LED system setup and the light cross-section area; Figure S2: Evaluation of virulence factors in MSSA strain under BL460nm irradiance; Figure S3: Normal QQ plot for test of normal distribution for the wound healing data; Figure S4: Total CFU count of MRSA recovered from the infected wound of five treatment groups; Table S1: Result of Shapiro-Wilk test for normality of data of treated infection wound model.
