# Supplementary material for: A Combination of Blue Light at 460 nm and H2O2 for the Safe and Effective Eradication of Staphylococcus aureus in an Infected Mouse Skin Abrasion Model
Source: Microorganisms. 2023 Dec 8;11(12):2946. doi: 10.3390/microorganisms11122946 (PMC10745725; doi:10.3390/microorganisms11122946)
Supplement: Supplementary file 1 [file microorganisms-11-02946-s001.zip › microorganisms-2719559-supplementary.pdf]

SUPPLEMENTARY MATERIALS

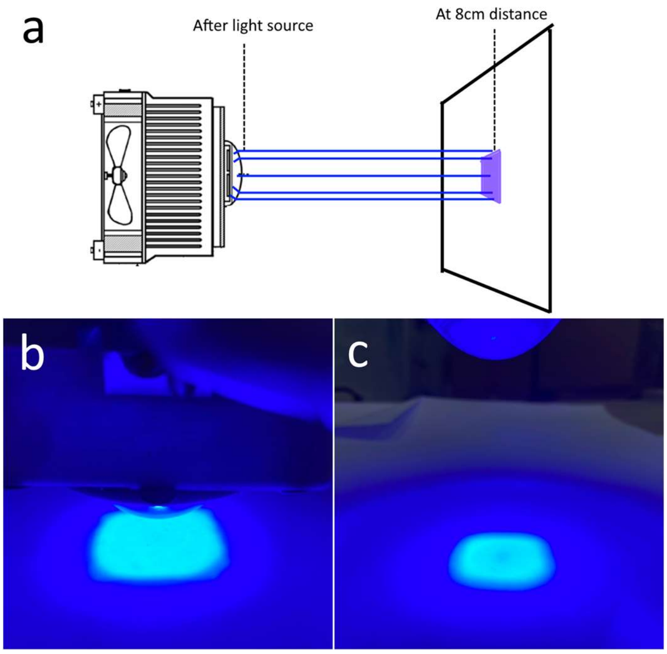

Figure S1: The LED system setup and the light cross-section area. a: Diagram of the light beam produced by the focus lens. b: Light cross-section area of the LED system after the light source. c: Light cross-section area of the LED system at an 8 cm distance.

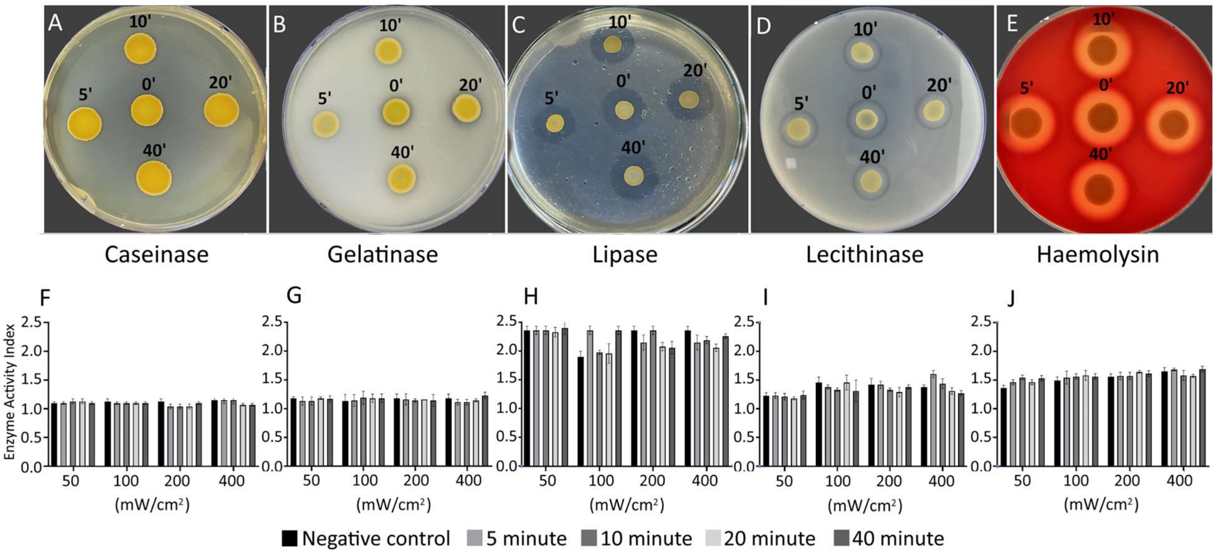

Figure S2. Evaluation of virulence factors in MSSA strain under BL460nm irradiance. The changes in virulent factors of the bacterial cells were examined at different light intensities (50, 100, 200, and 400 mW/cm^2) and different lightning durations (5, 10, 20, and 40 min). A and F: Caseinase, B and G: Gelatinase, C and H: Lipase, D and I: Lecithinase D, E and J: Heamolysin. Graphs in F-J were plotted from data of four replicates. Data are expressed as mean and standard deviation (SD). No statistical significance was found from the results of ANOVA and *post-hoc* Turkey testing for all the data.

Table S1. Result of Shapiro-Wilk test for normality of data of treated infection wound model

Test for normal distribution	*Negative*	*H_2O_2*	*BL460nm*	*FC*	*Combined*
Shapiro-Wilk test					
W	0.9570	0.9132	0.9522	0.8916	0.8685
P value	0.7813	0.3775	0.7335	0.2423	0.1457
Passed normality test (alpha=0.05)?	Yes	Yes	Yes	Yes	Yes
P value summary	ns	ns	ns	ns	ns
Number of values	8	8	8	8	8

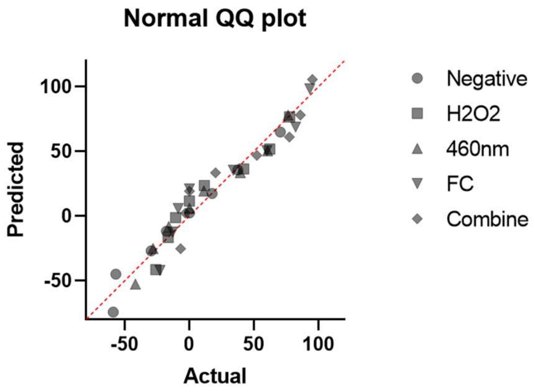

Figure S3: Normal QQ plot test of normal distribution for the wound healing data. The test was performed using Graphpad Prism 9.5.2 for Window operating system.

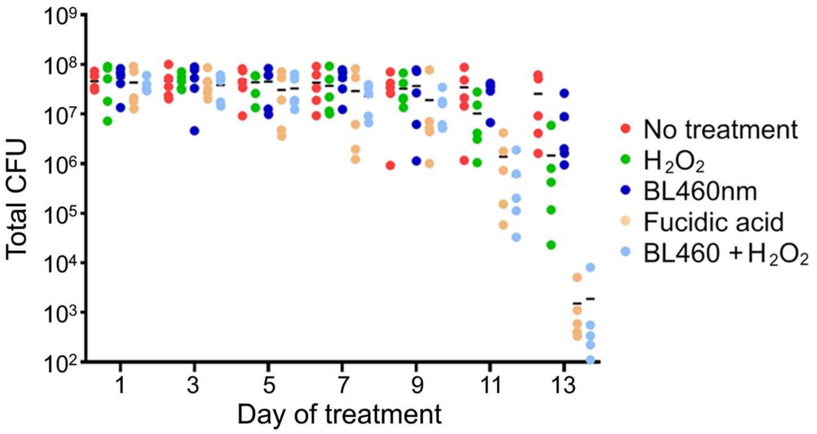

Figure S4. Total CFU count of MRSA recovered from the infected wound of five treatment groups. A sterile cotton swab pre-wetted with 5 μL saline solution (0.9%) was swabbed on wound area and resuspended in 200 μL saline solution. 100 μL of the sample was then diluted and spread on a Baird-Parker agar plate to count the total CFU available on the infection wound. Each dot represented the CFU count from each animal.
